# Transcriptome, intestinal microbiome and histomorphology profiling of differences in the response of Chinese sea bass (*Lateolabrax maculatus*) to *Aeromonas hydrophila* infection

**DOI:** 10.3389/fmicb.2023.1103412

**Published:** 2023-02-24

**Authors:** Chao Pan, Yanran Zhu, Kaixin Cao, Juexian Li, Siyu Wang, Jiahua Zhu, Xiaoman Zeng, Heqian Zhang, Zhiwei Qin

**Affiliations:** ^1^Center for Biological Science and Technology, Advanced Institute of Natural Sciences, Beijing Normal University, Zhuhai, China; ^2^College of Education for the Future, Beijing Normal University, Zhuhai, Guangdong, China; ^3^Faculty of Art and Science, Beijing Normal University, Zhuhai, Guangdong, China

**Keywords:** transcriptome, 16S rRNA, intestinal microbiota, immune response, interaction, histological damage

## Abstract

The Chinese sea bass (*Lateolabrax maculatus*) is an important aquaculture fish, but diseases caused by *Aeromonas hydrophila* have led to severe economic losses to the aquaculture industry in recent years. To date, only a few studies have focused on the relationship between the intestinal immune response and changes in intestinal microbes by *A. hydrophila* infection. Here, we report the transcriptome and intestinal changes in infected sea bass. Histopathological results showed that severe steatosis and vacuolation occurred in the liver and that the intestinal villi and mesentery were seriously affected after infection. By extracting total RNA from intestinal tissue and studying the transcriptome profile, 1,678 genes (1,013 upregulated and 665 downregulated) were identified as significantly differentially expressed genes (DEGs). These genes are involved in many immune-related signalling pathways, such as the NOD-like receptor, C-type lectin receptor, and Toll-like receptor signalling pathways. Moreover, the intestinal microbes of sea bass changed significantly after infection. Interestingly, at the genus level, there was an increase in *Serratia*, *Candida arthromitus* and *Faecalibacterium* as well as a decrease in *Akkermansia* and *Parabacteroides* after infection. The results also indicated that some of the DEGs involved in the immune response were related to the genus level of intestinal microbiota. Finally, there was a relationship between gene expression patterns and the bacterial structure in the host intestine. Our study provides a reference for the study of the immune response and particular functions of intestinal microbes of sea bass after pathogen infection.

## 1. Introduction

Chinese sea bass (*Lateolabrax maculatus*) is of commercial importance to China. However, in highly intensive farming environments, the emission of fish manure and other pollutants leads to environmental deterioration and the occurrence of aquatic animal diseases, especially in cage culture (Chen et al., 2018). Among those diseases, infection caused by bacterial pathogens has a serious impact on the cultivation of Chinese sea bass, which restricts the sustainable and healthy development of the sea bass cultivation industry.

*Aeromonas hydrophila* is a human-animal-aquatic comorbid conditional pathogen of aquatic animals that is widely distributed in various foods, soils, seawater and freshwater (Palanikani et al., 2020, Zhou et al., 2020). This gram-negative rod-shaped pathogen not only causes public health problems by carrying a variety of virulence factors (Igbinosa and Okoh, 2012) but also causes several pathological features in aquatic animals, including tail and skin rot and haemorrhagic septicemia (Abowei and Briyai, 2011; Mzula et al., 2019). Studies have shown that *A. hydrophila* enters fish *via* damaged skin or gills (Chu and Lu, 2008), destroys the intestinal mucus layer (Schroers et al., 2009) and induces macrophage apoptosis, consequently making the intestinal mucosa less effective against this pathogen (Shao et al., 2004). *A. hydrophila* is characterized by a wide area of damage and high morbidity and mortality, causing serious economic losses to the aquaculture industry (Harikrishnan and Balasundaram, 2005; Ge et al., 2011; Aboyadak et al., 2015).

The fish intestine has been shown to be significantly immunocompetent according to previous work on the structure of intestine-associated lymphoid tissue and other intestinal cell populations (Hart et al., 1988; Gomez et al., 2013). On the other hand, intestinal microbes play an important role in the growth and development, nutritional metabolism and immune resistance of the host (Round and Mazmanian, 2009). Therefore, maintaining the balance of the intestinal microbiota is crucial for the regulation of fish growth and immune function (Zhou et al., 2020). *A. hydrophila* infection of the intestine often causes host enteritis, and its infectivity depends on its bacterial toxins and metabolites competing with resident microorganisms in the host intestine (Ahishali et al., 2007; Song et al., 2014). The infection is often accompanied by mixed infection of other pathogens, and its pathogenicity may be related to changes in the composition and structure of the host intestinal microbiota (Qi et al., 2019). It is now believed that pathogenic infections interfere with the link between fish and their intestinal microbiota. Therefore, changes in the diversity of the fish intestinal microbes can be examined to assess their health and identify the response after bacterial infection (Xiang et al., 2010).

Unfortunately, there is a paucity of high-quality and complete genome annotation of the Chinese sea bass, which has limited the studies of host–pathogen interactions. However, in recent years, transcriptome sequencing has allowed the identification of immune-related genes (Zhao et al., 2016). RNA-seq high-throughput sequencing is different from traditional double-deoxygenation sequencing techniques and allows parallel sequencing of a large number of nucleic acid molecules simultaneously, resulting in a large amount of data (Li et al., 2013; Logares et al., 2014; Weidong et al., 2015). In addition, 16S rDNA sequencing has become a common method to reveal the composition of microbiota in intestinal content samples from aquaculture fish (Zhou et al., 2011; Ghanbari et al., 2015; Wang et al., 2018). The above methods are widely used to depict the immune mechanism of fish, which are exemplified in studies of Nile tilapia (*Oreochromis niloticus*) (Xiong et al., 2022), European eel (*Anguilla anguilla*) (Xiong et al., 2022), and Japanese sea bass (*Lateolabrax japonicus*) (Xiang et al., 2010).

In this study, the immune response and changes in the intestinal microbiota of Chinese sea bass 24 h after infection with *A. hydrophila* were analyzed using histopathological sections, transcriptome sequencing and 16S rRNA sequencing. Our results provide a basic model for further investigation of the intestinal immune mechanism of infected Chinese sea bass.

## 2. Materials and methods

### 2.1. Ethical statement

All experiments were conducted under the national regulations on the use of laboratory animals of China and approved by the ethics committee of laboratory animals of Beijing Normal University.

### 2.2. *Lateolabrax maculatus* and culture facility

Healthy Chinese sea bass (400 ± 50 g) were obtained from Zhuhai Fengwo, Co., Ltd. in Zhuhai, Guangdong Province, PR China. Thirty sea bass were allocated equally into two groups acclimating in two water tanks (153 × 120 × 55 cm) equipped with aerated filtered brine (1%) for 1 week. Filtering sand barrels were used for filtration with a flow velocity of 1,000 l/h, and fish were reared in fresh and oxygenated water (water temperature, 25 ± 0.5°C; DO, 6 ± 0.3 mg/L; pH, 7.5 ± 0.1). The rearing conditions of the sea bass remained unchanged throughout the experiment. Fish were anaesthetized using eugenol before they were humanely killed, and their samples collected.

### 2.3. *Aeromonas hydrophila* infection and sampling

*A. hydrophila* culturing and collection were performed according to Banerjee C’s study (Banerjee et al., 2012). For infection, the concentration of *A. hydrophila* was adjusted to 1 × 10^7^ CFU/mL. Eugenol (30 mg/L) was used to anaesthetize fish before injection. The infection group of sea bass was intraperitoneally injected with 500 μl of bacterial fluid. Another group of sea bass was intraperitoneally injected with 500 μL PBS as a control. Twenty-four hours after injection, the intestinal tissues and contents of fish in the control group and infection group were collected and frozen in a liquid nitrogen tank. The intestine of each fish was removed from the abdominal cavity and the mid-intestinal cavity. A total of 16 samples from 8 sea bass comprising the control group and infection group were collected for histological examination. The liver and intestine of sea bass in both groups were fixed with paraformaldehyde fixation solution.

### 2.4. Histopathologic investigation

The liver and intestines mentioned above were collected. The tissue was further dehydrated with alcohol and soaked in xylene. Then, the tissues were embedded in paraffin wax, sliced to a thickness of 4–6 μm, and stained with haematoxylin and eosin (H&E) to observe pathological changes (Mamun et al., 2022). The sections stained with HE were processed according to a standard protocol (Zhou et al., 2023), and the paraffin histological sections were examined under an upright optical microscope (Nikon ECLIPSE E100).

### 2.5. Total RNA extraction, cDNA library construction and sequencing

Intestines were obtained from the sea bass in the two Groups 24 h after infection. Total RNA of the two groups was extracted using the TRIzol method. RNA degradation and contamination were monitored on 1% agarose gels. RNA concentration was measured using a Qubit^®^ RNA Assay Kit in a Qubit^®^ 2.0 Fluorometer (Life Technologies, CA, United States). A total amount of 1 μg RNA per sample was used as input material for the RNA sample preparations. Sequencing libraries were generated using the NEBNext^®^ UltraTM RNA Library Prep Kit for Illumina^®^ (NEB, United States) following the manufacturer’s recommendations, and index codes were added to attribute sequences to each sample. The clustering of the index-coded samples was performed on a cBot Cluster Generation System using TruSeq PE Cluster Kit v3-cBot-HS (Illumina) according to the manufacturer’s instructions. After cluster generation, the library preparations were sequenced on an Illumina HiSeq platform, and 125 bp/150 bp paired-end reads were generated. High-throughput transcriptome sequencing was completed by Wuhan Metware Biotechnology Co., Ltd.

### 2.6. Raw data cleaning, *de novo* assembly and gene annotation

The original data measured by the Illumina HiSeq high-throughput sequencing platform were removed from the adapter and reads containing poly-N and of low quality, and unigenes were obtained through sequence splicing. Then, unigene sequences were compared to annotated proteins, including proteins in the Gene Ontology (GO), Clusters of Orthologous Groups (COG), Eukaryotic Orthologous Groups (KOG), and Kyoto Encyclopedia of Genes and Genomes (KEGG) databases.

### 2.7. Validation of DEGs by qRT–PCR

To verify the reliability of the RNA-seq data, qRT–PCR was conducted to quantify the expression levels of 7 representative DEGs related to complement pathways and defense responses. The primers with high amplification efficiency for these genes are listed in Supplementary Table S1. After evaluation with a NanoDrop One, RNA extracted from the fish intestines was reverse-transcribed to synthesize cDNA (Takara, Shanghai, China). qRT–PCR was performed with AceQ^®^ Universal SYBR Green qPCR Master Mix (Vazyme, Nanjing, China) in a QuantStudio(TM) 6 Flex System (Applied Biosystems, America). The reaction system and conditions of qRT–PCR were all according to the manufacturer’s instructions. The qRT–PCR program was as follows: initial denaturation step at 95°C for 5 min, 40 cycles of denaturation at 95°C for 10 s, annealing and extension at 60°C for 30 s. Each assay was conducted with *β-actin* as the internal reference. The data were quantified relative to the internal reference using the 2^−∆∆Ct^ method and represented the mean ± standard deviation of three replicates (Zhang et al., 2018).

### 2.8. High throughput sequencing of the 16S rRNA gene

Nine intestinal contents (three contents are merged into one pooled sample) from each group were randomly collected from water tanks 1 day after injection for high-throughput sequencing of the 16S rDNA. Total genomic DNA from pooled intestinal content samples of both groups was extracted using the CTAB/SDS method. DNA concentration and purity were monitored on 1% agarose gels. According to the concentration, DNA was diluted to 1 ng/μL using sterile water. 16S rRNA genes of distinct regions (V3-V4 hypervariable region) were amplified using specific primers 515F [5’-GTGCCAGCMGCCGCGGTAA-3′] and 806R [5’-GGACTACHVGGGTWTCTAAT-3′] with the barcode. All PCRs were carried out with 15 μl of Phusion^®^ High-Fidelity PCR Master Mix (New England Biolabs). The same volume of 1X loading buffer (containing SYBR Green) was mixed with the PCR products, and electrophoresis was performed on a 2% agarose gel for detection. PCR products were mixed in equidensity ratios. Then, the mixed PCR products were purified with a Gel Extraction Kit (Qiagen, Germany). Sequencing libraries were generated using the TruSeq^®^ DNA PCR-Free Sample Preparation Kit (Illumina, United States) following the manufacturer’s recommendations, and index codes were added. The library quality was assessed on the Qubit@ 2.0 Fluorometer (Thermo Scientific) and Agilent Bioanalyzer 2100 system. Finally, the library was sequenced on an Illumina NovaSeq platform, and 250 bp paired-end reads were generated. The 16S rRNA sequencing service was completed by Wuhan Metwell Biotechnology Co., Ltd.

### 2.9. Statistical and bioinformatics analysis

Clean data were obtained by filtering the off-machine data, and the reference sequences were obtained by splicing clean reads with Trinity. After comparing the above statistics, the mapped data were obtained and used to perform differential expression analysis according to the expression levels of genes in different groups. For screening differentially expressed genes, | log2Fold Change | > =1 and FDR < 0.05 were chosen as the standards, after which functional annotation and enrichment of expression level analysis were performed.

Splicing and quality control were carried out on the off-board data obtained by sequencing to acquire effective data, namely, clean data. To study the diversity of the species composition of each sample, OTU clustering was conducted based on the effective fragments of all samples, and species annotation was performed according to the OTU sequence. Complexity and diversity analyses were performed on the samples. MetaStat, Simper and LEfSe were selected to conduct analysis of intergroup species differences for the purpose of further study of the differences in community structure among different groups. Differential expression between the control and treatment groups was determined utilizing DESeq2 v1.22.1. The STRING database was used to perform protein interaction analysis of the differentially expressed genes, and a network was drawn accordingly. Pearson correlation analysis was used for the combined 16S and transcriptome analyses to form heatmaps and network interaction diagrams.

## 3. Results

### 3.1. Histopathological changes after *Aeromonas hydrophila* infection

**Figure 1:**
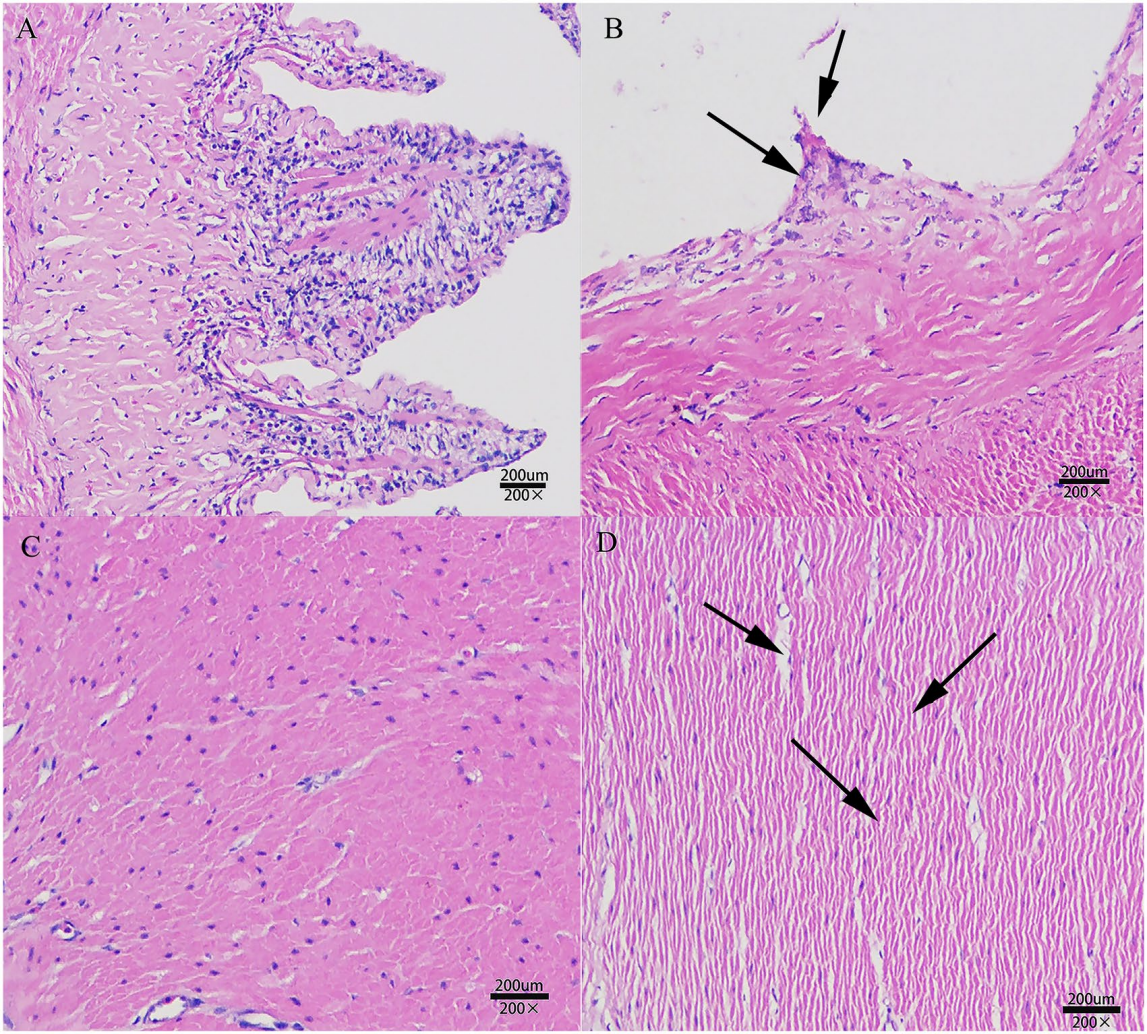
Histopathological changes in the intestine of the control and *Aeromonas hydrophila* groups. **(A)** The intestinal villi of the control group. **(B)** The intestinal villi and mesentery in the *A. hydrophila* group showed serious shedding (thick arrows). **(C)** The intestinal muscle layer cells of the control group. **(D)** The intestinal muscle layer cells of the *A. hydrophila* group. Thick arrows = loose arrangement. Thick arrows = vacuolated hepatocytes.

To more intuitively observe the changes in intestinal and liver tissue structure as well as cell morphology, we examined the histopathological changes after *A. hydrophila* infection. In the intestine tissue, the intestinal villi of the control group had an intact structure, the cells revealed normal morphology, and the intestinal muscle layer cells were arranged in a regular pattern (Figures 1A,C). In contrast, the intestinal villi and mesentery in the *A. hydrophila* group showed severe shedding (thick arrows in Figure 1B), the intercellular space was significantly increased, and the intestinal tissue was loose (thick arrows in Figure 1D). Moreover, the liver of the control group possessed a complete structure with normal hepatocyte (HC) morphology, and a normal structure of hepatic sinusoids (HS) was observed in the livers of control fish and fish in the *A. hydrophila* group (Supplementary Figure S1). However, the hepatocytes in the *A. hydrophila* group were severely vacuolated, and fat droplets accumulated in the cytoplasm, squeezing the nucleus to one side (thick arrows in Supplementary Figure S1B).

### 3.2. Differentially expressed genes after infection

**Figure 2:**
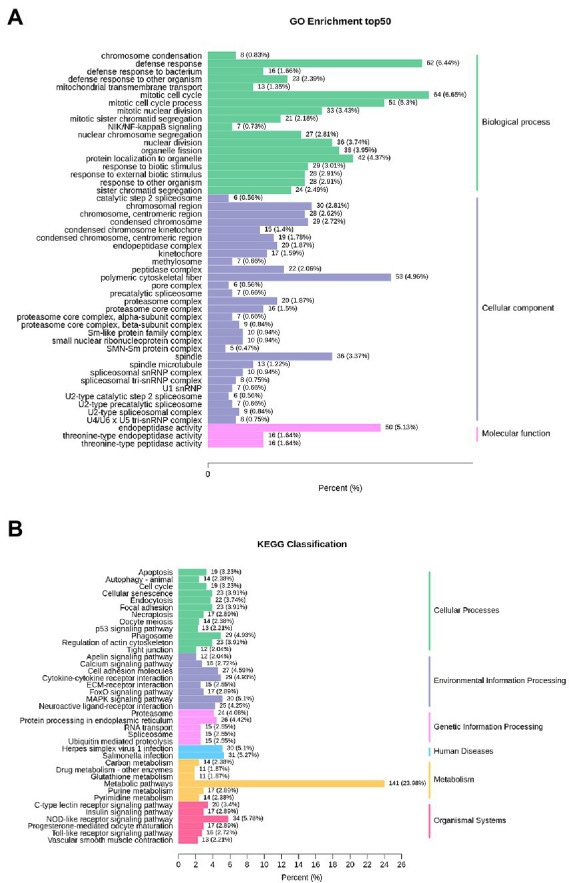
DEG analysis after infection with *Aeromonas hydrophila*. **(A)** Volcano plot of DEGs. **(B)** The biological process after infection by GO enrichment analysis.

To analyse the difference in gene levels between the infected and control groups, intestinal tissues were collected after injection for transcriptome analysis. The results showed that a total of 71.15 GB of clean data after sequencing for the two groups were obtained. The sequencing reads were mapped to the reference genome of Chinese sea bass, with mapping ratios ranging from 91.01 to 94.13%. Using StringTie v1.3.4d for new gene prediction, 2,718 novel genes were discovered. The corrected *p value* FDR < 0.05 and |log2fold change| ≥ 1 were used as the thresholds for significant differential expression. In total, 1,678 genes were differentially expressed in intestinal tissues; 1,013 upregulated and 665 downregulated genes were identified. Visualization of the screening results of differentially expressed genes (DEGs) through a volcano plot (Figure 2A) helped to determine the distribution. Many upregulated genes mediate or directly participate in the immune and inflammatory response, such as *IL-1β*, *IkB*, *ASC*, *TNFα*, *IL-8*, *TLR5*, *LBP*, *TLR5*, *IL-6R*, *PTX3*, and *C7*. Among them, several DEGs encoding pattern recognition receptors (PRRs) were significantly upregulated, including *NLRC3*, *TLR5*, and *LGP2*. Other DEGs, such as *C6*, *C7*, and *C4*, involved in the complement classical pathway were also highlighted. The sequencing data derived from the intestinal tissues of *L. maculatus* were stored in the NCBI Sequence Read Archive database under BioProject accession number PRJNA841263.

### 3.3. Related KEGG and GO pathways of DEGs

**Figure 3:**
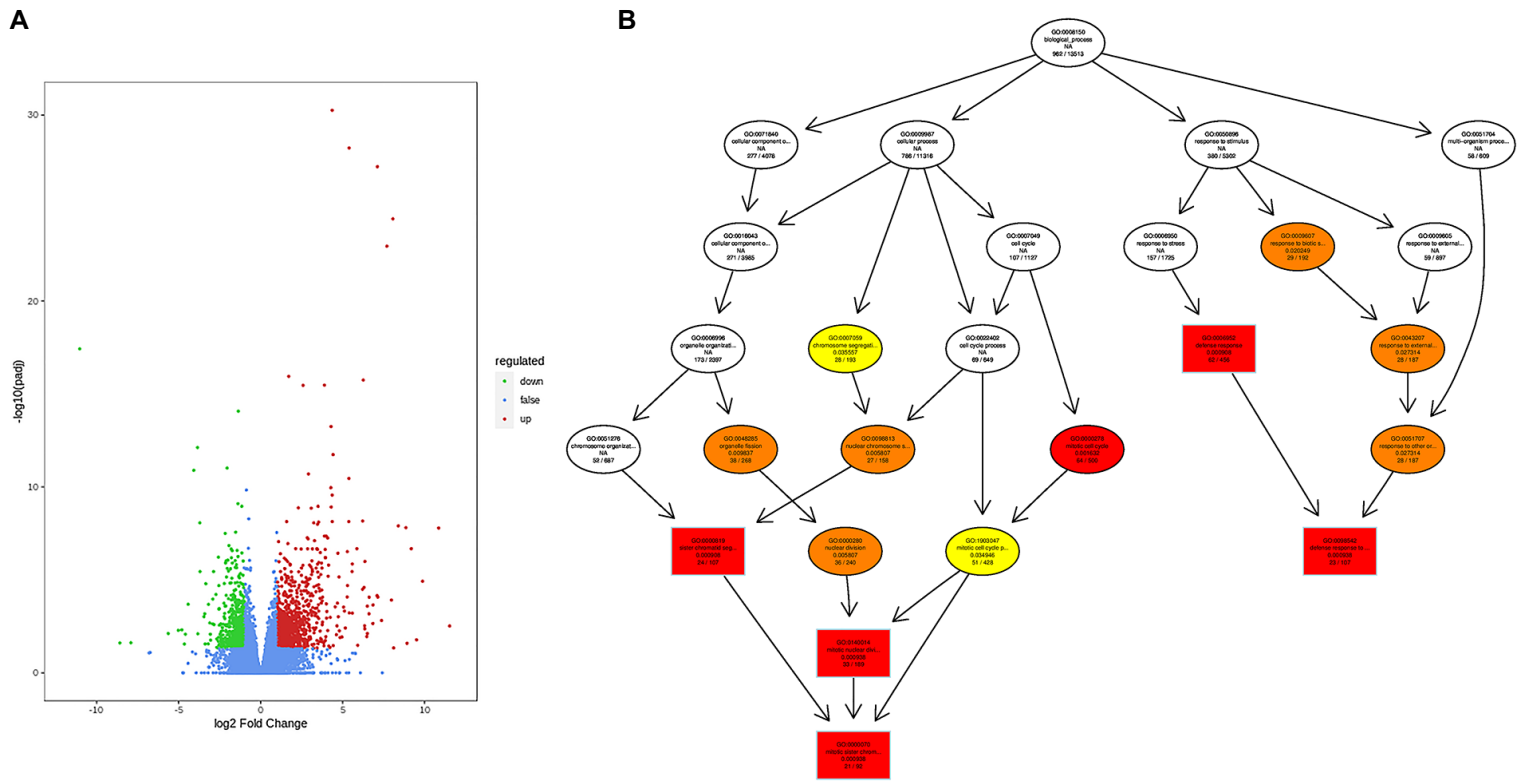
Enrichment column diagram of DEGs after infection with *Aeromonas hydrophila.* **(A)** GO enrichment analysis of DEGs after infection. **(B)** KEGG enrichment analysis of DEGs after infection.

The enrichment of DEGs after infection was analyzed using GO and KEGG databases for functional analysis. According to GO enrichment analysis, 18 genes were involved in biological processes, 29 genes in cellular components and 3 genes in molecular functions. The top 50 GO terms with the lowest q-value in the enrichment analysis are shown in Figure 3A. The most significantly enriched terms were mitotic cell cycle, defense response, polymeric cytoskeletal fibres, and endopeptidase activity. The immune-related GO terms took part in biological processes (Figures 2B, 3A), such as defense response to bacteria, biotic stimulus, external biotic stimulus and other organisms.

KEGG enrichment analysis showed that multiple pathways associated with inflammatory stress and the immune response were enriched with DEGs. The proteasome, the NOD-like receptor signalling pathway, pyrimidine metabolism, the phagosome, riboflavin metabolism, and protein export were enriched with most DEGs (Figure 3B). Some pathways directly participate in immune processes, such as the NOD-like receptor, C-type lectin receptor, Toll−like receptor and cytosolic DNA-sensing signalling pathways (Supplementary Figure S2). After infection with *A. hydrophila*, the expression levels of proinflammatory cytokine genes, proapoptotic genes, pathogen recognition-related genes and innate immune response activation-related genes were significantly upregulated (Supplementary Table S2).

### 3.4. DEGs were verified by qRT–PCR

To validate the accuracy of the DEG data, 7 genes related to complement pathways and defense responses from DEGs were selected for qRT–PCR analysis. As shown in Supplementary Figure S3, the expression of *C6*, *C7*, *IRAK4*, *LBP*, and *RIPK2* was significantly upregulated after infection with *A. hydrophila,* while the expression of *SIPA1L2* and *NFATC1* was significantly downregulated after infection. In general, the expression pattern of the above genes quantified by qRT–PCR was similar to that obtained in the RNA-seq analysis. Therefore, the analysis confirmed the reliability of the transcriptome data in RNA-seq.

### 3.5. Diversity analysis of intestinal microbes

**Figure 4:**
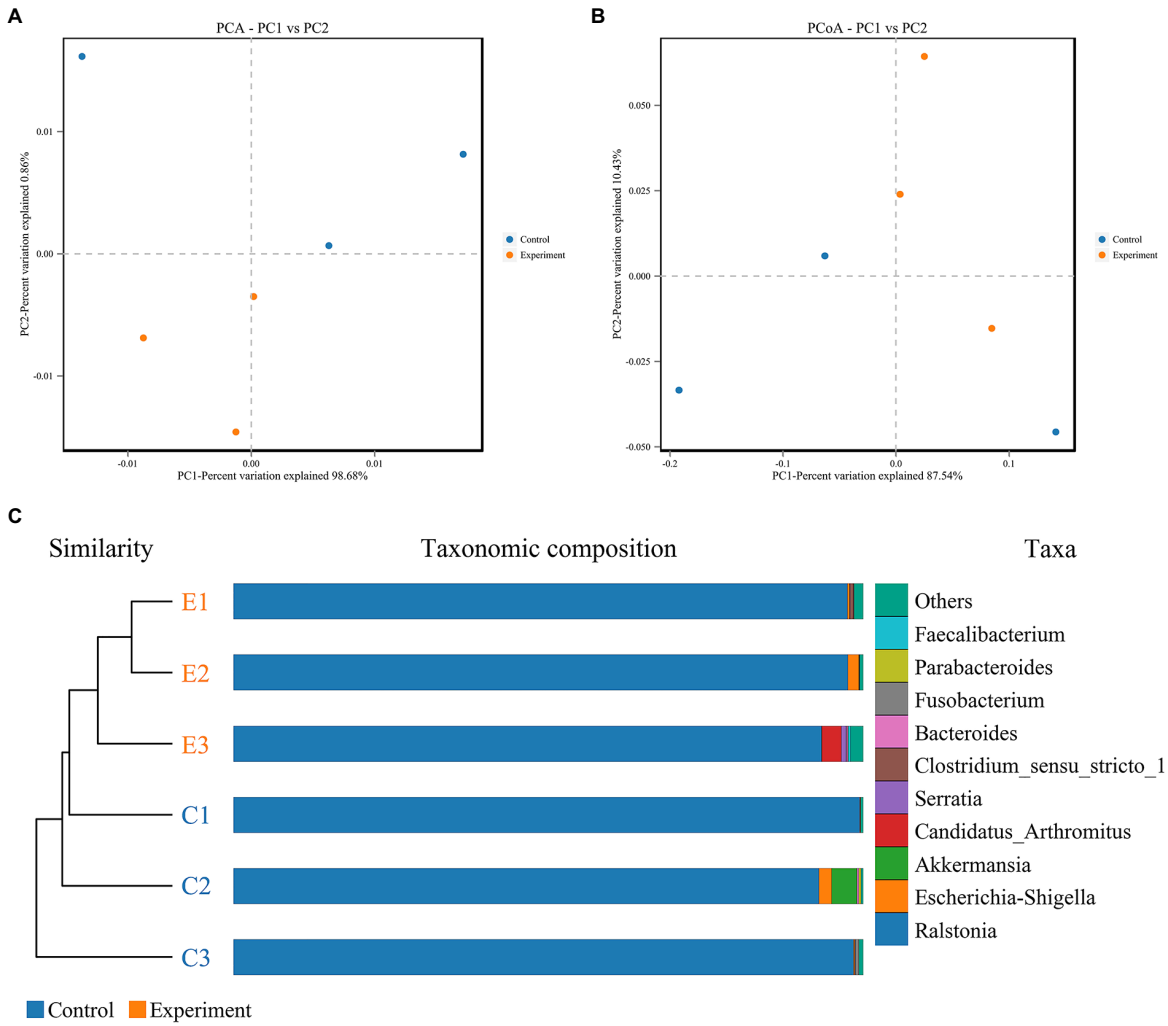
Beta diversity analysis of intestinal microbes of Chinese sea bass in the *Aeromonas hydrophila* infection and control groups. **(A)** Principal component analysis (PCA) of intestinal microbes. **(B)** Principal coordinates analysis (PCoA) of intestinal microbes. **(C)** UPGMA cluster tree analysis of intestinal microbes. The top right figure represents the top 10 species according to the species abundance in the table. Other species are classified as “Others,” and the species not annotated are unclassified.

After 16S rDNA sequencing, a total of 358,036 clean reads with an average of 59,673 clean reads per sample were obtained. Compared with the control group, the diversity of intestinal microbes in Chinese sea bass was significantly different in the *A. hydrophila* infection group. Principal component analysis (PCA) and cluster tree analysis indicated that the diversity of the intestinal microbiota changed significantly after infection. As shown in Figure 4A, a 98.68% distance in PC1 was observed in the PCA. Moreover, PCA was based on the distance matrix to find the principal component, and an 87.54% distance was observed in PC1 (Figure 4B). The cluster tree analysis displayed a close statistical distance between samples in the infected group (Figure 4C). The infected group also showed that, at the genus level, there was an increase in *Serratia*, *Candida arthromitus* and *Faecalibacterium* and a decrease in *Akkermansia* and *Parabacteroides*.

### 3.6. Changes in intestinal microbial composition after infection

**Figure 5:**
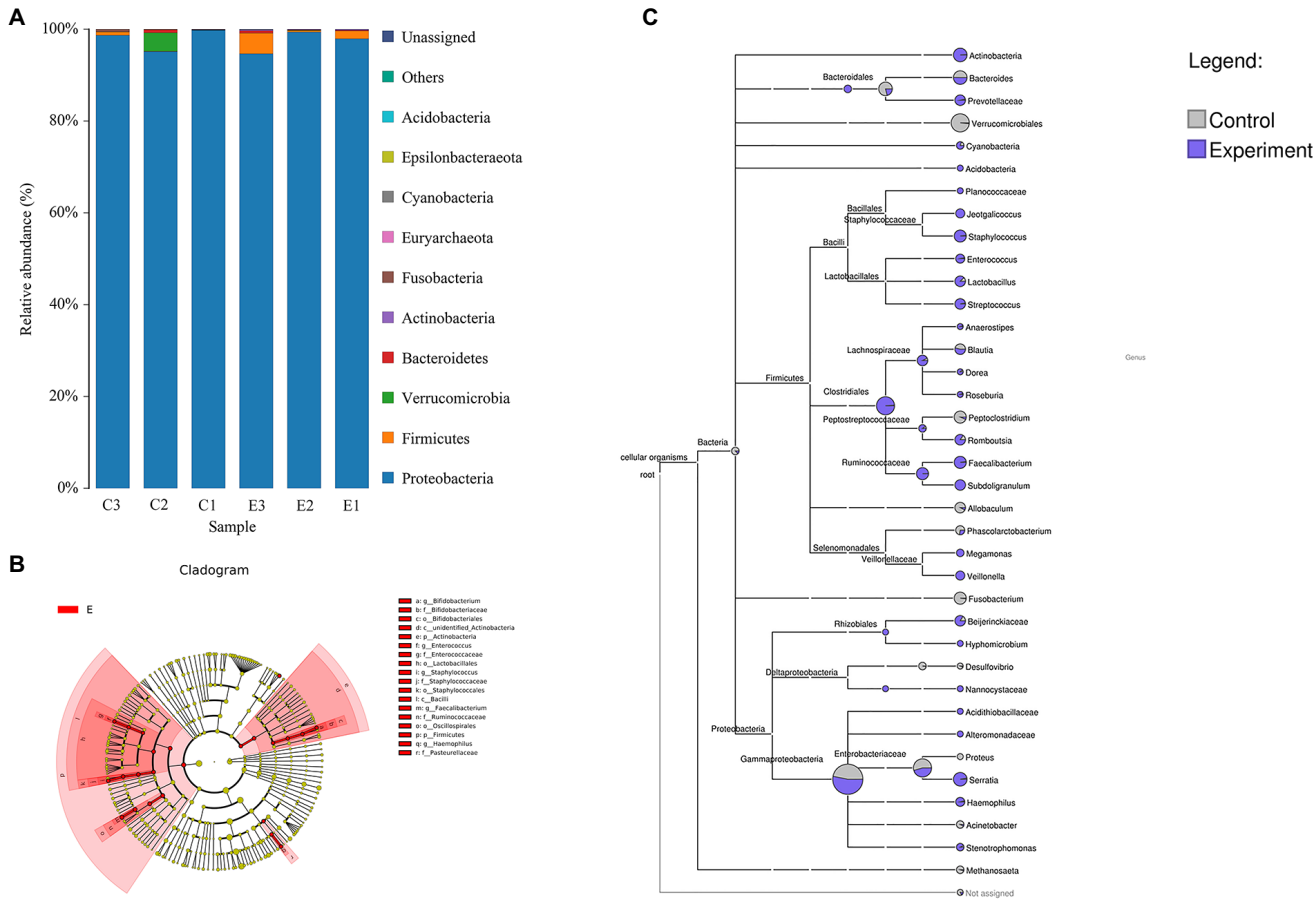
Changes in the intestinal microbial composition of Chinese sea bass after *Aeromonas hydrophila* infection. **(A)** Species annotation of intestinal microbes at the phylum level. **(B)** Taxonomic system relationship tree analysis of intestinal microbes. **(C)** Linear discriminant analysis (LDA) revealed significant differences in the intestinal microbiota. (LDA > 2, *p* < 0.05).

According to the species annotation results at the phylum level, Pseudomonadota, Bacillota, Fusobacteriota and Verrucomicrobiota were the main phyla in both groups (Figure 5A). The species that were significantly different in the intestinal microbial composition after infection are shown in Table 1 and were identified using Metastats. Among them, changes in the abundance of *Enterococcus*, *Bifidobacterium* and *Paeniclostridium* were the most apparent. The abundance information of intestinal microbes obtained by sequencing was returned to the database to generate a phylogenetic tree (Figure 5B). At the phylum level, we found that the abundance of Bacillota and Actinomycetes increased overall, and the abundance of Verrucomicrobia and Fusobacteria decreased significantly after infection. *Jeotgalicoccus* of Bacillota and *Hypomycetes* and *Nannocystaceae* of the Pseudomonadota phylum were annotated only in the infection group, while the abundances of *Peptoclostridium*, *Phascolarctobacterium, Allobaculum* and *Fusobacterium* of the Bacillota phylum and *Proteus* and *Acinetobacter* of the Pseudomonadota phylum decreased after infection. We then used line discriminant analysis to identify biomarkers with significant differences between the two groups, and all eligible biomarkers were from the infected group (*p* < 0.05, LDA values >2). Application of the LefSe method identified 5 significant differences at the family and genus levels separately and 6 significant differences at the species level. At the genus level, the relative abundances of *Faecalibacterium*, *Staphylococcus*, *Enterococcus*, *Bifidobacterium* and *Haemophilus* were differentially increased (Figure 5C).

**Table 1:** Significantly different species of intestinal microbes of Chinese sea bass after infection.

Species	Mean (control)	Variance (control)	Mean (infection)	Variance (infection)	value of *p*	*Q*-value
*Enterococcus*	1.32 × 10^−5^	5.23 × 10^−10^	3.15 × 10^−4^	1.02 × 10^−10^	0	0
*Bifidobacterium*	6.60 × 10^−6^	1.31 × 10^−10^	6.60 × 10^−4^	2.71 × 10^−8^	1.56 × 10^−3^	5.52 × 10^−2^
*Paeniclostridium*	1.98 × 10^−5^	1.18 × 10^−9^	1.55 × 10^−4^	1.35 × 10^−9^	3.11 × 10^−3^	7.36 × 10^−2^
*[Ruminococcus]_torques_group*	3.30 × 10^−5^	3.27 × 10^−9^	2.08 × 10^−4^	5.24 × 10–9	6.10 × 10–3	1.08 × 10^−1^
*Anaerostipes*	8.71 × 10^−6^	2.28 × 10^−10^	9.47 × 10^−5^	3.14 × 10^−9^	1.15 × 10^−2^	1.63 × 10^−1^

### 3.7. Differentially expressed genes related to the composition of bacterial communities

**Figure 6:**
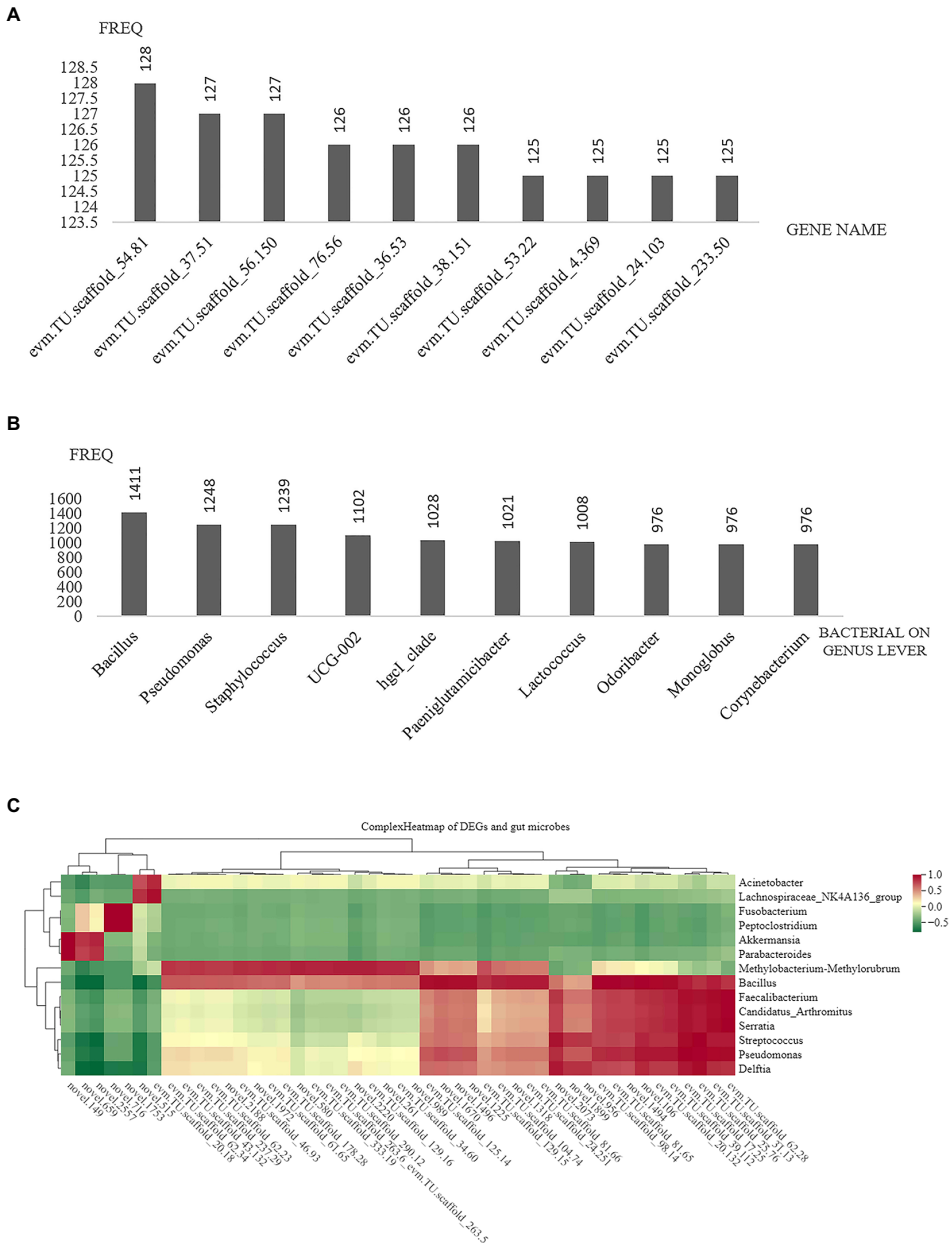
Relationship between differentially expressed genes and microbial community composition at the genus level. **(A)** Top 10 DEGs related to intestinal microbes. **(B)** Top 10 bacterial genera related to DEGs. **(C)** Heatmap of DEGs and intestinal microbes.

Pearson correlation was used to deduce the relationship between microbial community composition and DEGs at the genus level (Supplementary Table S3). A total of 30,291 pairs were explored, containing 1,672 genes and 239 genera (correlation >0.8, *p* < 0.05). Figure 6A shows the top 10 differentially expressed genes with genus-level relationships with intestinal microbes. Among them, some were involved in immune responses, such as cell adhesion molecule 1 (*Cadm1*) and thrombospondin-4-B (*thbs4b*). Other genes are involved in a variety of biological functions. Sodium-and chloride-dependent GABA transporter 2-like isoform X1 (*Slc6a13*) regulates GABA signalling termination through GABA uptake. Betaine-homocysteine S-methyltransferase 1 (*BHMT*) is involved in regulating homocysteine metabolism. Anoctamin-9 (*ANO9*) has calcium-dependent phospholipid scramblase activity. The top ten genera paired with DEGs were *Bacillus*, *Pseudomonas*, *Staphylococcus, Oscillospiraceae_UCG-002, hgcI_clade*, *Paeniglutamicibacter*, *Lactococcus*, *Odoribacter*, *Monoglobus*, and *Corynebacterium* (Figure 6B). Moreover, the genes with significant differences (|log2fold change| ≥ 5) were screened for association analysis with the top 30 genera of relative abundance, and a cluster heatmap and network interaction diagram were made accordingly (Figure 6C; Supplementary Figure S4).

## 4. Discussion

Chinese sea bass (*L. maculatus*) is an important fish species that has great economic value. As an aquaculture marine fish, it is widely distributed in China’s coastal areas (Wang et al., 2021). *A. hydrophila* is the main opportunistic pathogen that causes intestinal inflammation in aquaculture fish, resulting in great losses to fishery cultivation (Harikrishnan and Balasundaram, 2005; Tu et al., 2010; Zhou et al., 2020a). Although the pathogenesis remains unclear (Song et al., 2014), at present, many studies have focused on the mechanisms of the immune response and inflammatory diseases in fish after infection with pathogenic bacteria (Sudheesh et al., 2012; Liu et al., 2016; Charlie-Silva et al., 2019; Natnan et al., 2021). However, most of the studies aim to identify the DEGs of the host (Jiang et al., 2016; Yang et al., 2016; Ye et al., 2018; Xiong et al., 2022), and only a few studies focus on the changes in intestinal microbiota and the relationship with the transcriptomic data. In this study, we combined 16S rDNA and transcriptome sequencing to understand the changes in the intestinal transcriptome and microbes of infected Chinese sea bass, with the purpose of determining the relationship between gut microbiome composition and host gene expression.

### 4.1. Histopathology

The intestine is not only the vital organ of food digestion and nutrient uptake but also an important barrier against invading pathogens (Jutfelt et al., 2007). The liver plays a critical role in fish immunity in addition to being an important metabolic organ in fish (Thornton et al., 2017). Notable histopathologic changes have often been observed in the intestine and liver of fish after challenge with pathogenic bacteria. In our study, the intestinal villi and mesentery showed severe shedding after *A. hydrophila* infection, the intercellular space was significantly increased, and the intestinal tissue was loose. It was significantly different from that of the control group in terms of complete intestinal villi and tight arrangement of intestinal muscle cells. These pathological features are similar to a previous study that observed grass carp infected by *A. hydrophila* showed severe intestinal lesions, including intestinal villus shedding and heavy inflammatory cell infiltration (Song et al., 2014). After challenge with *A. hydrophila* for 1 day, the liver of sea bass had severely vacuolated hepatocytes, and fat droplets accumulated in the cytoplasm. These histopathological results indicate that liver cells are damaged and inflammation occurs, which was in agreement with Ahmed Hal, who stated that *A. hydrophila* causes liver vacuolar degeneration and pyknosis in hepatocytes (Hal and Manal, 2020). According to previous studies, extracellular products and toxins produced by *A. hydrophila* may cause severe necrosis in the liver (Afifi et al., 2000; Laith and Najiah, 2014).

### 4.2. Differentially expressed genes

Transcriptome profiling based on high-throughput sequencing technology has been recognized as an important method to assess transcriptional responses to different challenging conditions (Sun et al., 2012; Jiang et al., 2016). After infection, 1,678 DEGs with significant fold changes were screened, some of which were related to immune and inflammatory responses, and their functions after infection need to be further explored. The innate immune system plays a key role in the early recognition of invading pathogens (Neves et al., 2009). It activates innate immune cells through pattern recognition receptors (PRRs), achieves phagocytosis of pathogens and initiates signal transduction (Thompson et al., 2011). The combination of pattern recognition receptors on the surface of immune cells and pathogenic bacteria triggers an inflammatory response, activates related signalling pathways, and stimulates the production of interferons and other cytokines (Muñoz-Carrillo et al., 2019). In this study, several putative PRR molecules, including NOD-like receptor C3 (NLRC3), Toll-like receptor 5 (TLR5) and ATP-dependent RNA helicase DHX58 (LGP2), were significantly upregulated. RIG-I-like receptor (RLR) family proteins all contain the DExD/H box-containing RNA helicase domain and the C-terminal domain (CTD) (Yoneyama and Fujita, 2009). LGP2, a member of the RLR family of proteins, also plays an important role in the adaptive immune response, regulating T-cell survival and apoptosis through various pathways (Ireton and Gale, 2011; Ramos and Gale, 2011). Interleukins are important cytokines produced by immune cells and involved in immune regulation and inflammatory processes (Luzina et al., 2012). Recent studies have shown that large numbers of DEGs were found in the spleen of *Micropterus salmoides* after *A. hydrophila* infection, and many proinflammatory cytokines were largely upregulated after infection (Yuan et al., 2021). As we anticipated, several interleukins, including interleukin-1 beta (IL-1*β*) and interleukin-8 (IL-8), were induced after infection in this study. The complement system, an important component of the innate immunity of teleosts, mediates the recruitment of immune competent cells and the direct killing of pathogens (Boshra et al., 2006). Its activation and activity are finely regulated by various proteins in the body. In our data, several complement components, such as complement component 6 (C6), complement component 7 (C7) and complement component 4 (C4), were significantly upregulated. These results suggest that the complement classical pathway is activated after infection.

### 4.3. Enrichment analysis of DEGs

The enrichment of DEGs after infection was based on gene annotation, Gene Ontology (GO) and KEGG pathway analysis. The KEGG classification analysis showed that the most upregulated genes were enriched in metabolic pathways, and several metabolic processes were enriched in GO enrichment analysis. These results are similar to the enrichment analysis of DEGs in largemouth bass after challenge with *A. hydrophila*, which revealed that fish metabolism was severely affected by infection (Yuan et al., 2021). Moreover, further analysis showed that a number of upregulated genes were enriched in immunity-related pathways, especially the cytosolic DNA-sensing pathway, the Toll-like receptor signalling pathway, the RIG-I-like receptor signalling pathway and the NOD-like receptor signalling pathway. After infection, many DEGs are enriched in these pathways in other fish species (Xiong et al., 2022). A large number of upregulated genes were involved in both innate and adaptive immune pathways, which is similar to most previous studies (Patel et al., 2016; Wang et al., 2019; Mastrochirico-Filho et al., 2020). However, a number of genes associated with adaptive immunity in largemouth bass were significantly downregulated after infection, which may be inhibited by inflammation (Yuan et al., 2021). Our data suggested that *A. hydrophila* infection had a great impact on the immune response of sea bass, which could be a factor leading to fish inflammation. The enrichment analysis suggested that after infection, the immune defense and proinflammatory pathways of sea bass were activated, and cytokine signal transduction and interaction responses had an effect on eliminating invading pathogens.

### 4.4. Changes in intestinal bacterial phylotypes

Intestinal microbes are involved in the regulation of host growth and development, nutrient metabolism, and immune defense (Backhed et al., 2005; Round and Mazmanian, 2009). At the same time, they may also serve as potential pathogenic sources to cooperate with other pathogenic bacteria to accelerate host infection (Ohland and Jobin, 2015, Zhang et al., 2018). Bacterial infections can affect the homeostasis of fish intestinal microbes, and it is important to understand the structure of gut microbial communities and analyse how they change and respond to different situations (Jung-Schroers et al., 2018; Deng et al., 2020; Zhou et al., 2020a; Yuan et al., 2021). Previous studies have found that the diversity and uniformity of the intestinal microbiota in fish is significantly decreased after infection with *A. hydrophila* (Zhou et al., 2011; Yuan et al., 2021). However, there was no significant decrease in the diversity and uniformity of the intestinal microbes of *L. maculatus* after infection, which may be related to the difference in bacterial concentration and duration of infection. PCA and UPGMA analysis based on four distance matrices were used to compare species diversity between different samples. Nevertheless, through UPGMA and PCA, we found that the intestinal microbes differed significantly between the control and infected groups. In previous studies, it was also observed that the intestinal microbiota after *A. hydrophila* challenge were separately clustered, and the distance in PC1 was smaller than our data (Zhang et al., 2018). The results indicated that the intestinal microbiota tended to have similar changes after infection, which was different from the control group. To reveal the microbial diversity fluctuation after infection, we next collected samples at each time interval.

Pseudomonadota, Bacillota, Fusobacteriota and Verrucomicrobiota were the main phyla in both groups, which is consistent with the results of a previous study on the dominant phylum of intestinal microbes in marine fish (Jones et al., 2018). Some dominant microbial phyla interact closely with each other and participate in maintaining the microbial community structure (Segata et al., 2011; Kehe et al., 2019). From the taxonomic tree, the fluctuations of dominant microbes affected the changes in intestinal microbial composition. The annotation results showed that the abundances of a large number of genera changed after infection, and some of those changes were significant, suggesting that infection severely disrupted the homeostasis of the host intestinal microbiota. The majority of intestinal microbiota are obligate anaerobes. As facultative anaerobes, Pseudomonadota can affect the homeostasis of anaerobic environment in relation to the oxygen concentration in the gut, and eventually driving the composition of intestinal microbiota towards the dominant direction of facultative anaerobes (Litvak et al., 2017). The results showed that the abundance of Pseudomonadota decreased significantly after infection, indicating that the composition of the intestinal microbiota and oxygen balance changed. LEfSe analysis, an analytical tool that emphasizes statistical significance and biological relevance, was used to find biomarkers with significant differences after *A. hydrophila* challenge. The results showed five biomarkers at the genus level and six at the species level with higher abundance after infection. The relationship between the change in its abundance and the infection process needs to be further explored.

### 4.5. Relationship between the DEGs and bacterial community composition in the intestine

According to previous studies, the intestinal microbiota modulates the expression of some genes in intestinal epithelial cells (Larsson et al., 2012; Broderick et al., 2014; Richards et al., 2019; Shi et al., 2021). The intestinal microbiome of *Drosophila melanogaster* has significant impacts on host gene expression and intestinal structure, likely due to microbial products and elicitors (Broderick et al., 2014). The genes altered by the presence of the intestinal microbiota are widely distributed, including genes involved in the immune response, metabolism and developmental pathways (Broderick et al., 2014). The intestinal microbiota regulates the expression of a large number of genes in the small intestine and fewer genes in the colon, and MyD88 is required for microbiota-induced colonic expression of the antimicrobial genes Reg3b and Reg3g in the epithelium. The important role of the human intestinal microbiota in regulating host gene expression and manipulating the microbiome composition may be useful in future treatments (Richards et al., 2019). Other studies have also confirmed that the intestinal microbiota and the host intestinal epithelium should be regarded as an interrelated entity (Shi et al., 2021) and that the percentages of *Aeromonas* and *Rosemonas* and the differential expression of IL12 are associated with the disease resistance of Huanghe carp to new strains (Su et al., 2021). Our results indicated that some of the top 10 differentially expressed genes related to the genus level of intestinal microorganisms were involved in the immune response, such as cell adhesion molecule 1 (CADM1) and thrombospondin-4-b (thbs4b). Cell adhesion factors are involved in cell recognition, cell activation and signal transduction and the molecular basis for a series of important physiological and pathological processes, such as the immune response and inflammation (Ren et al., 2010). In response to peripheral nerve injury, thrombospondin-4 is significantly upregulated in the dorsal spinal cord (Frolova et al., 2012). According to the pairing results, *Bacillus*, *Pseudomonas*, and *Staphylococcus* were related to the differential expression of genes in intestinal tissue. In the intestines of *Cyprinus carpio L*. and Nile tilapia, *Bacillus* is considered to have a strong antagonistic effect on *A. hydrophila* (Mulyani et al., 2018; Kuebutornye et al., 2020). The clustering heatmap and network map obtained from association analysis suggest that intestinal microbes may influence gene expression in the host intestine and that dominant species or bacterial structure may reflect the host’s defense against pathogen invasion.

## Data availability statement

The data presented in the study are deposited in the NCBI Sequence Read Archive database under BioProject repository, accession number PRJNA841263.

## Ethics statement

The animal study was reviewed and approved by all experiments were conducted under the national regulations on the use of laboratory animals of China and approved by the ethics committee of laboratory animals of Beijing Normal University.

## Author contributions

CP, YZ, JL, KC, XZ, and JZ collected the samples and wrote the original draft. CP combined the analysis of transcriptome and microbiome sequencing data and discussed the results of intestinal microflora changes. SW analyzed the intestinal microbial composition. JL and KC discussed the results of the transcriptomic changes. XZ analyzed and discussed the results of the histopathological changes. CP and YZ performed the real-time qPCR experiment. ZQ and HZ supervised the project reviewed the original draft. All authors contributed to the article and approved the submitted version.

## Funding

This work was supported by Beijing Normal University *via* the Youth Talent Strategic Program Project (310432104) and Start-up Fund (111032103), teaching reform research project from Beijing Normal University (jx2022059), the College Youth Innovative Talent Program of Department of Education of Guangdong Province (2022KQNCX155), the start-up fund from National College Students innovation and entrepreneurship training program (X202119027050).

## Conflict of interest

The authors declare that the research was conducted in the absence of any commercial or financial relationships that could be construed as a potential conflict of interest.

## Publisher’s note

All claims expressed in this article are solely those of the authors and do not necessarily represent those of their affiliated organizations, or those of the publisher, the editors and the reviewers. Any product that may be evaluated in this article, or claim that may be made by its manufacturer, is not guaranteed or endorsed by the publisher.
