# Supplementary material for: Transcriptome, intestinal microbiome and histomorphology profiling of differences in the response of Chinese sea bass (Lateolabrax maculatus) to Aeromonas hydrophila infection
Source: Front Microbiol. 2023 Feb 24;14:1103412. doi: 10.3389/fmicb.2023.1103412 (PMC9998533; doi:10.3389/fmicb.2023.1103412)
Supplement: Supplementary file 1 [file Data_Sheet_1.PDF]

*Supplementary Material*

# Transcriptome, intestinal microbiome and histomorphology profiling of differences in the response of Chinese sea bass (*Lateolabrax maculatus*) to *Aeromonas hydrophila* infection

## Supplementary Table 1

Sequence information of primers for qRT-PCR.

| Primer No. | Primer name | Sequence (5' - 3') |
|---|---|---|
| 1 | C6-F | ACCAAGTTCTGCCTGTTTCCA |
| 2 | C6-R | CCCTGAGCTGTAGTAGTGCGTC |
| 3 | LBP-F | AGCGTTTCCCAGGTCTGATGATGA |
| 4 | LBP-R | AGGGTGGCGTTGGGTTGGATAG |
| 5 | NFATC1-F | CAACATGCGGGCCATGATCG |
| 6 | NFATC1-R | ACCATCCGCACTCGTGTGTT |
| 7 | RIPK2-F | GACCTGCGGCTGGACCTCTAACC |
| 8 | RIPK2-R | GACCCCTCATCACTCCTCTTCTCACA |
| 9 | SIPA1L2-F | GACAGCGGCATCGACACTAATCC |
| 10 | SIPA1L2-R | GGACATAATGGCAGAGGCGTAGC |
| 11 | C7-F | TGACAAAACGCCTCCCAACTC |
| 12 | C7-R | CTGCACTGCCCTCCGAAACTC |
| 13 | IRAK4-F | CGACTTGCTTGCTTGGAGGG |
| 14 | IRAK4-R | ACGCTCTCGTCAGCCCAAAG |

| | | |
|---|---|---|
| 15 | β-actin-F | CGTGCTGTCTTCCCCTCCA |
| 16 | β-actin-R | TCACCAACGTAGCTGTCCTTCTG |

**Supplementary Table 2** Partial of DEGs involved in signaling pathway related to immune response

| Gene name | Gene ID in transcriptome | $Log_2FC$ | Regulated | *p*-value |
|---|---|---|---|---|
| Toll−like receptor signaling pathway | | | | |
| *TLR5* | evm.TU.scaffold_129.16 | 7.15 | up | $1.16\times10^{-6}$ |
| *CASP8* | evm.TU.scaffold_255.49 | 1.54 | up | $1\times10^{-3}$ |
| *CD40* | evm.TU.scaffold_11.322 | 1.34 | up | $2.81\times10^{-3}$ |
| NOD-like receptor signaling pathway | | | | |
| *HSP90* | evm.TU.scaffold_1.108 | -1.52 | down | $1.06\times10^{-3}$ |
| *ASC* | evm.TU.scaffold_14.2 | 2.00 | up | $1.43\times10^{-4}$ |
| *IL-1β* | evm.TU.scaffold_287.11 | 4.55 | up | $5.43\times10^{-6}$ |
| *IRF3/7* | evm.TU.scaffold_4.273 | 2.71 | up | $1.17\times10^{-5}$ |
| C-type lectin receptor signaling pathway | | | | |
| *IκBα* | evm.TU.scaffold_137.4 | 3.24 | up | $1.79\times10^{-11}$ |
| *MALT1* | evm.TU.scaffold_6.267 | 1.41 | up | $1.22\times10^{-3}$ |
| *RELB* | evm.TU.scaffold_56.112 | 1.54 | up | $1.46\times10^{-3}$ |
| *Cox-2* | evm.TU.scaffold_12.30 | 1.67 | up | $4.31\times10^{-3}$ |

**Supplementary Table 3** Relationship between differentially expressed genes and microbial community composition on genus level

| Gene ID | NR | Chr | regulated | Correlation | P-value | Genus |
|---|---|---|---|---|---|---|
| evm.TU.scaffold_6.46 | CD44 antigen | 6 | Down | 0.828 | 0.042 | *Ralstonia* |
| evm.TU.scaffold_76.153 | Zinc finger protein 362-like isoform X1 | 4 | Down | 0.848 | 0.033 | *Ralstonia* |
| evm.TU.scaffold_19.80 | Ankyrin repeat and SOCS box protein 5-like isoform X1 | 14 | Down | 0.863 | 0.027 | *Ralstonia* |
| evm.TU.scaffold_188.45 | Growth/differentiation factor 10-like | 13 | Up | -0.872 | 0.024 | *Ralstonia* |
| novel.1379 | Unknown | 23 | Up | -0.831 | 0.040 | *Ralstonia* |
| novel.2442 | Uncharacterized protein LOC108881995(predicted) | scaffold_446 | Down | 0.814 | 0.049 | *Ralstonia* |
| evm.TU.scaffold_25.115 | Serum amyloid A | 1 | Up | 0.983 | 0.000 | *Pseudomonas* |
| evm.TU.scaffold_98.14 | Metalloreductase STEAP4 isoform X2 | 7 | Up | 0.955 | 0.003 | *Pseudomonas* |
| evm.TU.scaffold_24.251 | Unknown | 7 | Up | 0.944 | 0.005 | *Pseudomonas* |
| evm.TU.scaffold_25.76 | Haptoglobin-like isoform X1 | 1 | Up | 0.992 | 0.000 | *Pseudomonas* |

**Supplementary Fig. 1**

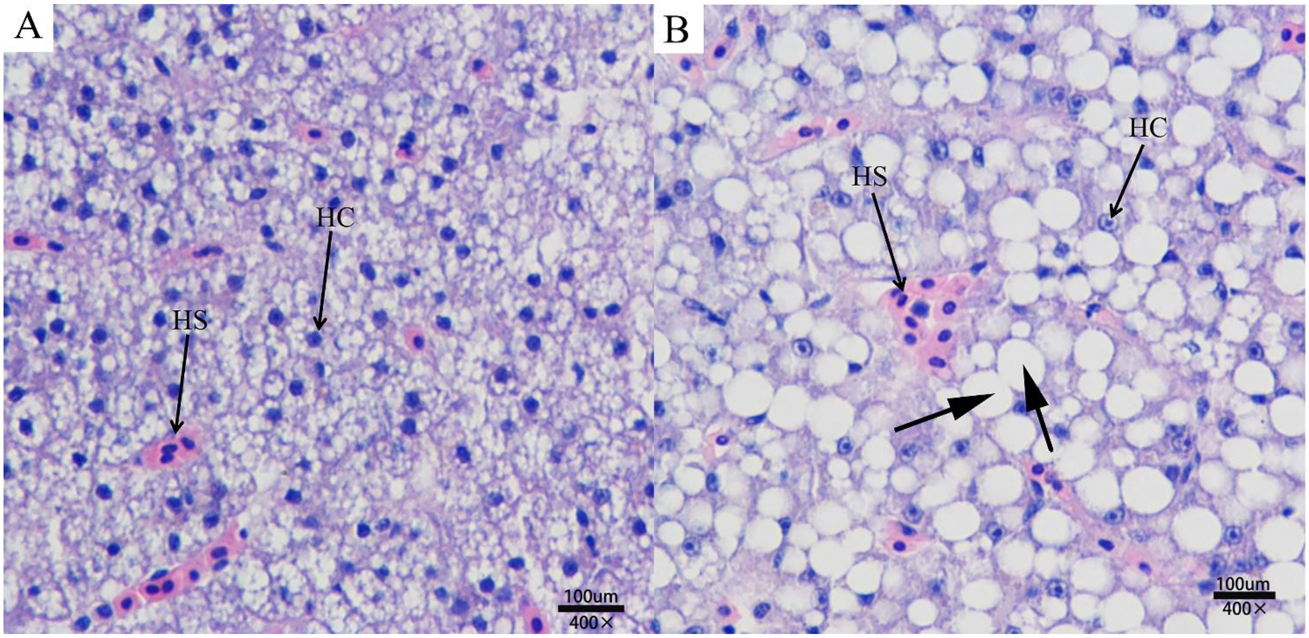


**Supplementary Fig.1** Histopathological changes in the liver of the control and *A. hydrophila* groups.

**Note:** A. The liver of the control group. B. The liver of the *A. hydrophila* group. Thick arrows= vacuolated hepatocytes, HS = hepatic sinusoid, HC = hepatocyte.

## Supplementary Fig. 2

A

B

C

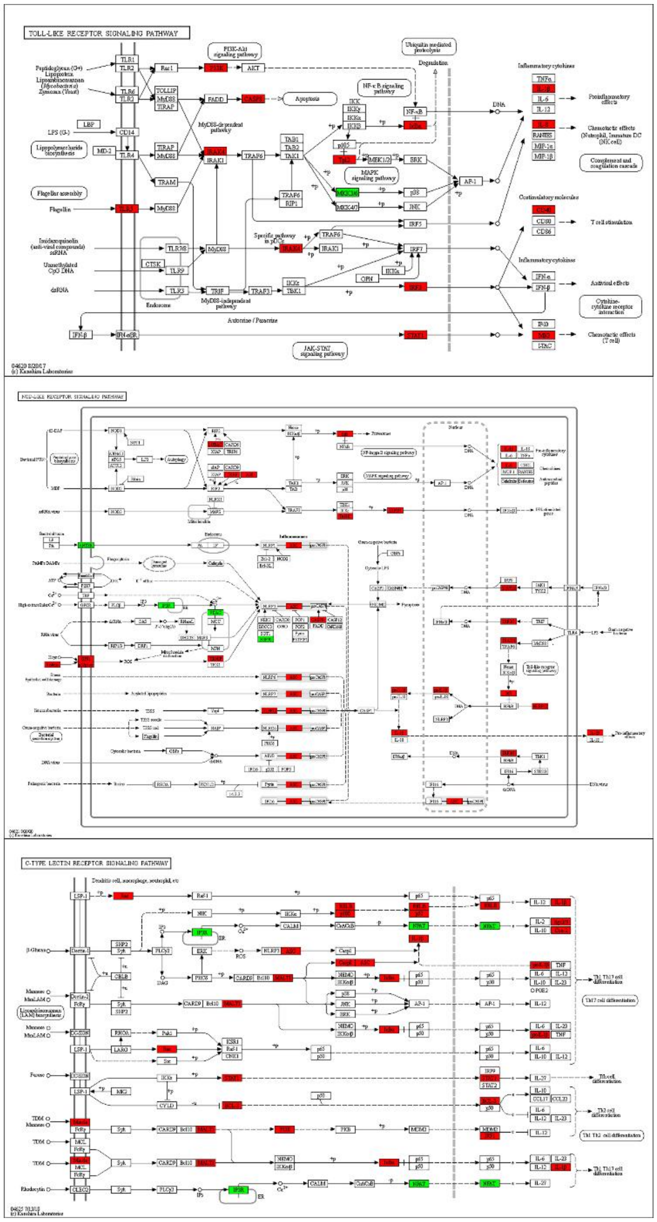

**Supplementary Fig. 2** KEGG pathways annotation of differentially expressed genes. (A) DEGs are annotated to the Toll-like receptor signaling pathway. (B) DEGs are annotated to the NOD-like receptor signaling pathway. (C) C-type lectin receptor signaling pathway. For the infected group, the red box labeled protein is related to the up-regulated gene, and the green box labeled protein is related to the down-regulated gene. According to the differences of samples, the differential expression of genes related to metabolic pathway was mainly studied.

**Supplementary Fig. 3**

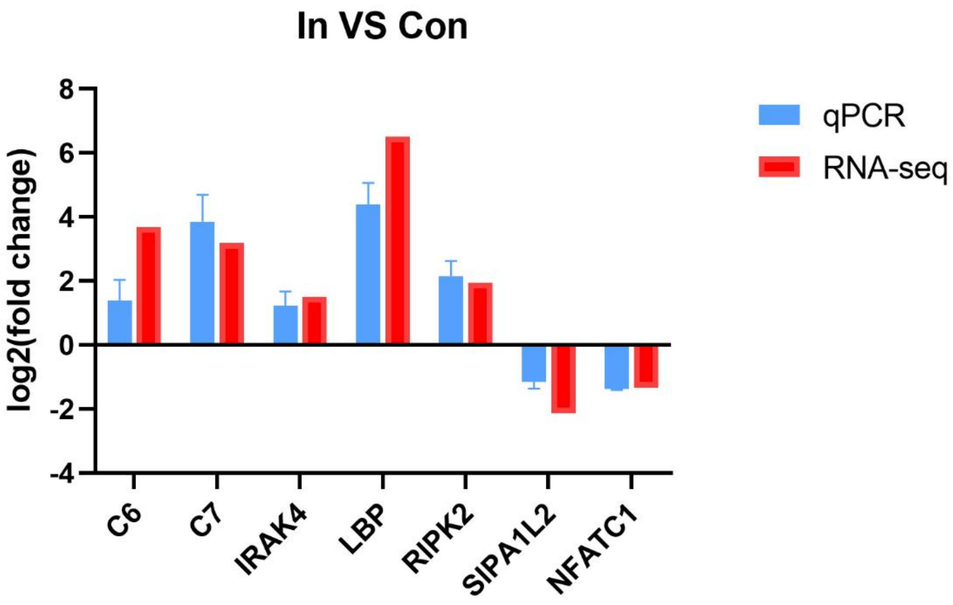


**Supplementary Fig. 3** Validation of RNA-seq data by qRT-PCR analysis. X-axis, gene names; Y-axis, $log_2$(fold change) in gene expression. The relative expression of seven DEGs related to complement pathways and defense responses were determined by qRT-PCR with the *β-actin* as the internal reference.

## Supplementary Fig. 4

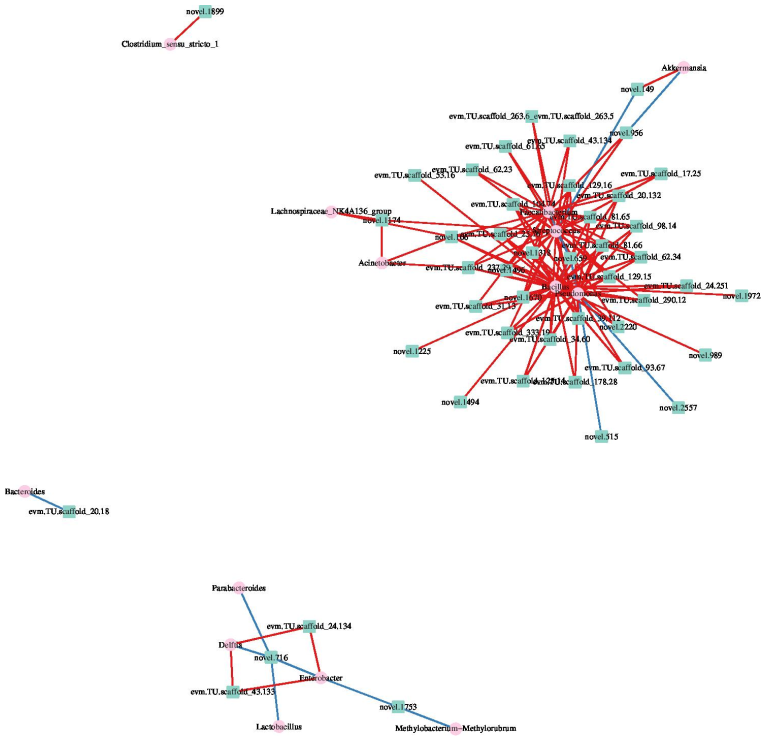


**Supplementary Fig. 4** Network interaction diagram of association analysis with the top 30 genera and significantly differentially expressed genes. The genes with significant differences (|log2foldchange| ≥ 5) were screened for association analysis with the top 30 genera of relative abundance.
